# Restoring Engagement in Digital Self-Control Tools Using Nudge Reconfiguration Prompts: Quasi-Experimental Study

**DOI:** 10.2196/85349

**Published:** 2026-04-28

**Authors:** Awen Kidel Peña--Albert, Sandy Ingram, Yasser Khazaal, Léo Litrico, Juan Carlos Farah, Denis Gillet

**Affiliations:** 1 École Polytechnique Fédérale de Lausanne, Rte Cantonale, Lausanne, 1015, Switzerland, 33 0782662496; 2 School of Engineering and Architecture of Fribourg (HEIA-FR), HES-SO, Fribourg, Switzerland; 3 Addiction Medicine, Department of Psychiatry, Lausanne University, University Hospital of Lausanne, Lausanne, Vaud, Switzerland

**Keywords:** digital self-control tools, digital well-being, smartphone addiction, nudge, self-determination theory, behavior change, user engagement, mobile apps, self-report, screen time

## Abstract

**Background:**

Digital self-control tools (DSCTs) have emerged as technological interventions to address excessive smartphone usage and promote digital well-being. However, these tools face persistent challenges with user attrition and sustained engagement, compromising their long-term effectiveness. Current literature lacks an understanding of how observable behavioral indicators, as opposed to self-reported measures, are associated with user engagement and readiness to change in DSCTs.

**Objective:**

This study addresses three research questions (RQs): (RQ1) whether prompting passive DSCT users to reconfigure nudges increases subsequent user-nudge interaction, (RQ2) how engagement evolves over time and what behavioral divergence emerges between accepting and rejecting users, and (RQ3) whether observable in-app behavioral indicators are more strongly associated with intervention acceptance than traditional self-reported measures.

**Methods:**

We conducted a quasi-experimental study (N=252) targeting users who had disabled nudges. Participants were randomly assigned to receive a prompt to reconfigure their nudge settings during daily check-ins (n=138, experimental group) or to a control condition (n=114, no intervention). The experimental group was further classified into acceptance and rejection subgroups based on their response to the intervention. Data collection included DSCT configuration logs, usage-triggered nudge logs, and self-reported questionnaire responses. We analyzed user-nudge interaction ratios using difference-in-differences with permutation tests (RQ1) and nudge configuration parameters and manual app blocking using independent-samples *t* tests with Cohen *d* (RQ2) and compared behavioral indicators against self-reported measures using *t* tests and chi-square tests (RQ3).

**Results:**

Of the experimental participants, 46% (63/138) accepted the nudge reconfiguration prompt. Post intervention, the acceptance subgroup’s 7-day average user-nudge interaction ratio increased from 29.7% to 58.5% (peak of 65% on day 1), a significant increase even after controlling for the temporal decline observed in the control group (difference-in-differences=+36.3 percentage points, *P*<.001). The rejection subgroup’s decline was not significantly different from the control group’s decline (*P*=.82). The acceptance subgroup showed preexisting behavioral indicators of higher readiness to change, including 21.53% shorter consecutive usage thresholds (*P*=.03) compared to the rejection subgroup, with a directionally consistent but nonsignificant difference in cooldown length (+20.56%). Behavioral divergence in consecutive usage thresholds widened post intervention, with Cohen *d* increasing from −0.47 to −0.67 (*P*=.002). Acceptance subgroup participants demonstrated a significantly lower tendency to select leisure-oriented daily goals (15.6% vs 26.2%; chi-square *P*=.001, Cramer *V*=0.13). Self-reported measures of screen time goals and scrolling regret were not significantly associated with intervention acceptance (*P*>.10).

**Conclusions:**

Observable in-app behavioral indicators, rather than self-reported measures, effectively differentiate intervention receptiveness. Study results suggest that effective DSCT design should incorporate adaptive strategies that recognize and respond to users’ readiness to change, as evidenced by their in-app behaviors, while preserving autonomy. Such systems are likely to outperform static interventions or designs that rely solely on self-reported preferences.

## Introduction

### Background

Concerns about digital well-being, the equilibrium individuals maintain in their relationship with mobile devices [1,2], have become a prominent public health issue as smartphones increasingly mediate daily activities [3,4]. Social media platforms, driven by engagement-maximizing recommendation algorithms [5,6], create environments in which users’ conscious intentions to moderate their digital consumption compete against scientifically designed engagement mechanisms [7], contributing to excessive screen time and associated mental health concerns [8]. As awareness of these dynamics has grown, so has interest in interventions that support users in self-regulating their digital behaviors without removing the underlying technologies from their lives.

In response to these emerging public health challenges, researchers and developers have explored technological solutions to promote digital well-being and self-regulation. These attempts led to the emergence of digital self-control tools (DSCTs) designed to help users monitor and regulate their digital activities [9,10]. These tools embody a paradoxical approach: using technology to regulate technology by using digital interventions that modify users’ decision-making environments [11]. DSCTs operate across various platforms and contexts, from browser extensions that modify YouTube’s user interface by removing recommendation feeds [12] to mobile apps designed to address excessive smartphone usage [10].

Central to DSCTs is the concept of the nudge: an element of choice architecture that predictably alters behavior without restricting options [13]. Increasingly applied to digital environments as “digital nudging” [14], DSCTs employ various nudge strategies, from friction-based approaches that intercept every app launch [15] to threshold-based nudges that activate only when usage exceeds a self-configured limit [9,10]. The latter approach preserves user autonomy by operating passively until triggered by actual behavior, but its effectiveness depends critically on configuration: thresholds set too high may never trigger, rendering the tool inert, whereas settings perceived as too restrictive may provoke abandonment [16]. This makes the user’s willingness to configure and maintain meaningful nudge parameters, rather than the tool’s technical capabilities, a central determinant of DSCT effectiveness.

One Sec, a mobile DSCT, exemplifies the friction-based approach: it intercepts every attempt to open a target application with a deliberative interface combining messaging, temporal friction, and the option to cancel [15]. While effective over a 6-week period [15], the high-friction nature of such interventions generates user resistance [16], highlighting a broader tension in DSCT design between intervention intensity and long-term retention.

Empirical evidence underscores this tension: studies on DSCTs have yielded mixed results, with one study finding that such interventions lacked perceived effectiveness [17]. While a meta-analysis found that control tools may be linked to reduced smartphone use, it did not address whether disconnection alone fosters digital well-being [11]. Current implementations of these tools face substantial limitations, particularly in maintaining sustained user interaction [18], which is a common issue for digital and smartphone-based interventions [19-21]. The prevalent approach of implementing prescheduled interventions often results in a misalignment between users’ initial motivations and their subsequent behavioral patterns, sparking a feeling of helplessness or annoyance in users and ultimately compromising the long-term effectiveness of these tools [16]. The primary challenge lies in developing interventions that not only initiate but also maintain behavioral change while preserving user autonomy throughout the process.

User retention is a well-documented problem across mobile health apps [22], with motivational decline identified as a primary abandonment factor [23]. Within DSCTs, specifically, excessive or unreliable interventions might precipitate tool abandonment. Current literature identifies 2 critical research priorities: implementing effective blocking strategies and encouraging users to maintain properly configured nudge settings that generate meaningful interventions over time. Our study focuses on the latter: users who have disabled their nudge configurations are of particular interest because they represent a subgroup that initially engaged with the DSCT but subsequently became passive, potentially signaling declining readiness to self-regulate.

Understanding what drives users to actively configure and engage with DSCTs requires a theoretical framework for motivation and behavior change. Self-determination theory (SDT) posits that sustained behavior change depends on the satisfaction of 3 basic psychological needs: autonomy, competence, and relatedness [24]. When applied to technology design, SDT suggests that autonomy-supportive interfaces, those that allow users to set their own parameters rather than imposing restrictions, foster more lasting engagement [25,26]. Complementary to SDT, the transtheoretical model frames behavior change as progressing through stages of readiness, from precontemplation to maintenance [27]. In the DSCT context, users who actively configure nudge parameters may be at a different readiness stage than those who have disengaged, and observable in-app behaviors could serve as proxies for these otherwise latent motivational states [28].

Despite growing research on DSCT effectiveness, a critical gap remains: current literature relies primarily on self-reported measures to assess user engagement and motivation, yet the behavioral factors, observable through configuration logs and usage data, that determine whether users continue to engage with nudges over time remain underexplored. There is limited work investigating whether in-app behavioral indicators can predict intervention receptiveness more effectively than traditional self-reported measures. Identifying such indicators is essential for developing adaptive interventions that not only initiate behavioral change but sustain it through continued user engagement.

### Research Questions

This study investigates whether prompting passive DSCT users to reconfigure their nudge settings can restore engagement and whether observable behavioral indicators are more strongly associated with intervention receptiveness than self-reported measures. Through a quasi-experimental protocol, we target users who have disabled nudges (or have set their usage threshold excessively high so that nudges are never triggered) and invite them to reconfigure their nudge settings. We address the following 3 research questions (RQs):

- RQ1: To what extent does prompting users to reconfigure nudges after deactivation increase subsequent user-nudge interaction and engagement?
- RQ2: How does user engagement with nudges evolve over time after a reconfiguration prompt, and what behavioral divergence emerges between accepting and rejecting users?
- RQ3: To what extent do users’ in-app behavioral indicators, including nudge configurations, usage-triggered interaction logs, and daily goal selections, serving as proxies for intent and readiness to change, reveal meaningful differences between users who accept and those who reject DSCT intervention prompts, in comparison with self-reported measures?

The findings aim to inform the design of more effective digital well-being interventions by identifying observable indicators of user engagement and readiness to change.

## Methods

### Digital Self-Control Tool

#### Tool Development

Detox [29] is an iOS app launched in November 2023 designed to foster sustainable digital well-being by implementing targeted app-blocking mechanisms. The app specifically targets excessive smartphone use over short durations, a behavior commonly associated with *doomscrolling*—the excessive consumption of short-form content on smartphone apps [30]. While the term originally emerged during the COVID-19 pandemic to describe the excessive consumption of negative news, it has since evolved in colloquial usage to refer more broadly to the inability to disengage from endless or infinite scrolling interfaces displaying short-form videos on social media [31].

The development of Detox followed an iterative methodology inspired by the lean startup model [32]. An initial prototype was created prior to conducting user interviews or engaging early adopters. Once the first version was released, usage patterns of early adopters were monitored, and approximately 50 in-depth user interviews were conducted to refine the product. Feedback from these interviews highlighted that Detox effectively mitigated excessive social media engagement, particularly patterns linked to doomscrolling, thereby guiding the app’s evolution to better meet user needs.

Over a 12-month period, Detox evaluated various behavioral modification strategies by adjusting the “friction coefficient” of smartphone app access, including social media access. This involved experimenting with restrictive interventions, such as blocking access to apps until the following day after excessive use, and more lenient approaches, such as only sending reminders in the form of notifications to stop scrolling. By November 2024, insights from more than 8000 users supported the creation of a theory-informed and empirically anchored framework of digital interventions, drawing on behavioral data, interviews, and SDT. These interventions were meticulously calibrated to address maladaptive social media interaction patterns to promote healthier digital habits.

#### Underlying Conceptual Framework

The theoretical underpinning of Detox’s intervention framework prioritizes user autonomy in self-regulation over the imposition of restrictive measures [24,33]. Grounded in SDT, Detox emphasizes 3 core psychological needs—autonomy, competence, and relatedness—which are widely recognized as critical components of apps designed to facilitate behavior change [24,26,34]. These constructs are conceptualized as follows:

- Autonomy: The need to perceive oneself as the originator of one’s actions, with behaviors aligned to personal values and interests.
- Competence: The need to feel proficient and effective in one’s endeavors. When competence is satisfied, individuals gain confidence in their ability to master tasks and attain desired outcomes, thereby bolstering motivation and engagement.
- Relatedness: The need to experience meaningful connections and a sense of belonging within interpersonal relationships. Fulfillment of this need fosters psychological well-being and sustains motivational processes.

According to SDT, the satisfaction of these 3 needs supports intrinsic motivation, promotes personal growth, enables sustained behavior change, and contributes to psychological well-being. This framework aligns closely with contemporary theories of behavioral modification that advocate for sustainable change [28]. By embedding these principles, Detox’s approach offers a theoretically robust foundation for addressing phone addiction while respecting individual agency.

#### Intervention Design

**Figure 1.**
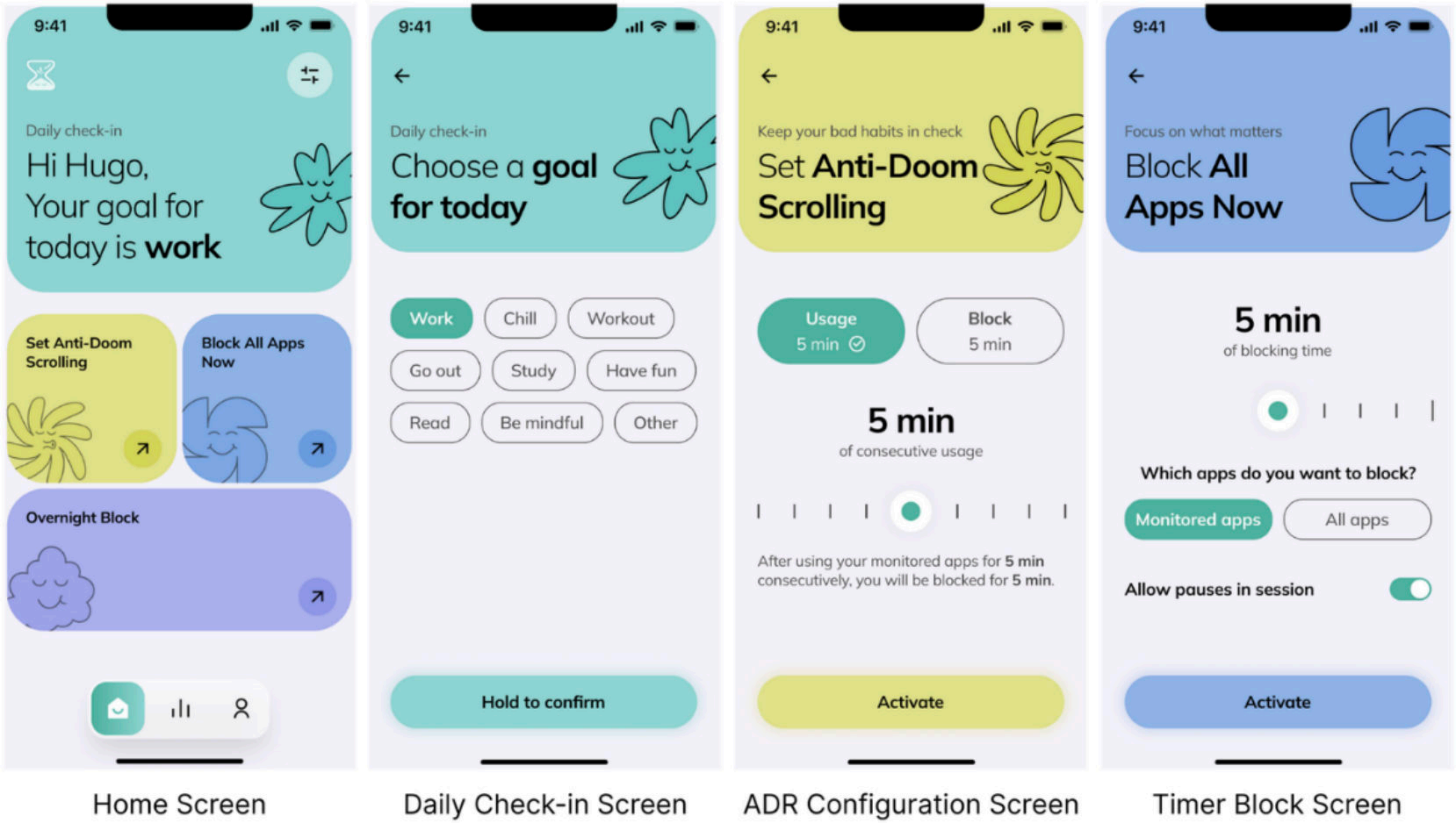
Main screens of the Detox app demonstrating the user interface design grounded in self-determination theory, featuring customizable intervention parameters that preserve user autonomy while facilitating digital self-regulation.

For each intervention, users maintain the agency to decide which apps they want to monitor. Detox implements 3 distinct categories of interventions, as illustrated in Figure 1.

##### The Daily Check-In

This serves as the primary forced intervention mechanism. Beginning at 4 AM daily, the system automatically restricts access to monitored apps until users complete their daily check-in within the Detox interface. During this process, users establish a daily objective (eg, “Chill,” “Work,” or “Study”), which informs subsequent intervention protocols throughout the day (Figure 1, second panel).

##### The Anti-Doomscrolling Reminder

This represents a user-configured nudging mechanism that is activated based on continuous usage patterns. The intervention is triggered when usage duration exceeds a user-defined threshold (eg, 5 min of continuous engagement), resulting in a temporary restriction period (eg, 1 min). Both the continuous usage threshold and restriction duration are customizable parameters determined by the user (Figure 1, third panel).

##### The Timer Block/Overnight Block

These functionalities enable temporal restriction of monitored apps for user-specified durations (Figure 1, fourth panel).

For clarity and consistency throughout the remainder of this paper, we will employ the following terminology: “Daily Check-In” will continue to be referred to as such; “The Anti-Doomscrolling Reminder” will be referred to as the “nudge feature”; and “The Timer Block/Overnight Block” will be referred to as “manual app blocking.” These designations will facilitate precise discussion of each intervention type and its respective effects on user behavior.

### Study Design

#### Participant Recruitment

The study population consisted of users from the DSCT user base who engage with the app through mandatory daily check-ins. Within this population, we distinguish between active users—those who demonstrate regular user-nudge interaction on a weekly basis by receiving nudges—and passive users—those who complete daily check-ins but do not receive any nudges. Our recruitment specifically targeted users who had disabled nudges prior to enrollment; that is, users who had previously configured nudge settings but subsequently deactivated them or set their consecutive usage threshold too high for nudges to be triggered.

The total DSCT user base comprised 472 daily check-in users: 252 (53.4%) were passive users meeting our eligibility criteria, whereas 220 (46.6%) were active users with regular nudge interactions and thus ineligible for this experiment. We successfully recruited all 252 eligible passive users through a rolling enrollment protocol over 3 days (November 22‐24, 2024). Participants in the experimental group (n=138) each received exactly 1 prompt to reconfigure settings during this period. This sample represents the maximum possible recruitment from the target population.

Participants were randomly allocated to 1 of 2 conditions: the experimental group (n=138, 54.8%), which received a prompt to reconfigure their nudge settings during their daily check-in, or the control group (n=114, 45.2%), which consisted of passive users who met the same eligibility criteria but received no intervention, serving as a baseline for comparison. Within the experimental group, those who accepted the prompt were subsequently classified as the acceptance subgroup, whereas those who declined formed the rejection subgroup.

Randomization between experimental and control groups was performed automatically at the device level, with allocation decisions executed independently for each participant. Although the study protocol targeted an equal distribution (50%) between groups, the observed imbalance resulted from the decentralized randomization process, which operated without global coordination and allowed for independent assignment variations across devices. No incentives were provided for participation or intervention acceptance; participants’ decisions to accept or decline the prompt were entirely voluntary and self-motivated.

#### Procedure

The experimental group (n=138) was presented with the intervention before completing their daily goal selection during the rolling enrollment window (November 22‐24, 2024). Based on their response, participants were classified into acceptance or rejection subgroups. Group assignment remained fixed throughout the study period.

The control group (n=114) completed their daily check-in without receiving any intervention, maintaining access to all application features.

Usage data were collected continuously for 5 weeks: 1 week before intervention and 4 weeks post intervention.

#### Intervention

**Figure 2.**
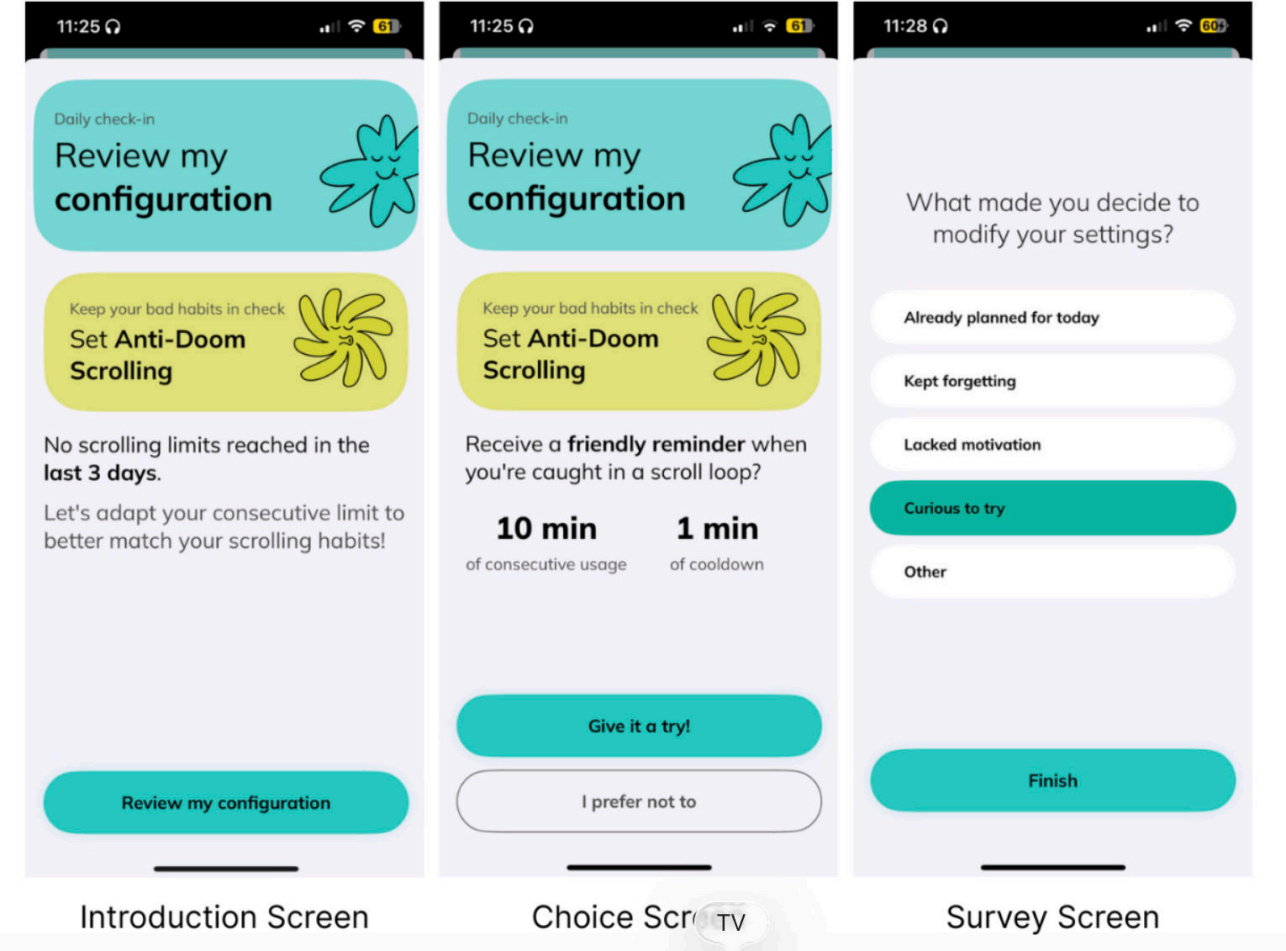
Experiment screens illustrating the quasi-experimental protocol for the nudge reconfiguration prompt, highlighting the preservation of user autonomy through voluntary participation and the systematic data collection framework.

The intervention consisted of 3 screens integrated into the daily check-in flow, as depicted in Figure 2. The detailed user journey of the experiment design can be found in Figure S1 in Multimedia Appendix 1. The intervention screens are as follows:

- Introduction screen: Presented before daily goal selection, this screen highlighted the user’s inactive nudge configuration with the message “Review my configuration” and prompted them to “Set Anti-Doom Scrolling.”

- Choice screen: Displayed a suggested nudge configuration of 10-minute consecutive usage followed by a 1-minute cooldown period. Users could accept (“Give it a try!”) or decline (“I prefer not to”) this configuration.
- Survey screen: A mandatory single-choice questionnaire captured the user’s rationale for their decision, with response options including “Kept forgetting,” “Looked motivating,” and “Other.”

Experimental participants maintained complete autonomy regarding intervention acceptance and subsequent settings adjustments. For those who accepted the prompt, the system suggested a 10-minute consecutive usage limit as a default reconfiguration option. This recommendation was designed to provide a reasonable, nonextreme starting point that would minimize potential user frustration [16] while encouraging engagement with the nudge system. Participants remained free to adjust their settings to higher or lower thresholds at any time following their initial reconfiguration, ensuring continued autonomy over their digital well-being preferences.

#### Data Collection and Instruments

Data collection was conducted through the Mixpanel analytics platform, which implemented event-based logging protocols to systematically record user interactions. We collected 2 categories of data: (1) DSCT configuration logs and usage-triggered nudge logs, which serve as observable behavioral indicators (used for RQ1, RQ2, and RQ3) and (2) self-reported questionnaire data from onboarding surveys and postintervention surveys (used for RQ3). Key metrics are defined below.

- User-nudge interaction ratio: The user-nudge interaction ratio represents the daily proportion of users in a given group for whom the nudge mechanism was effectively triggered (ie, the user reached the configured consecutive usage threshold and a “nudge reached” event was logged), relative to the total number of users in that group. This metric reflects actual behavioral intervention based on real usage, not merely whether the nudge feature was enabled in the app configuration.
- Consecutive usage threshold: A user-configured nudge parameter (in min) representing the continuous screen time threshold on monitored apps before a nudge is triggered.
- Cooldown length: A user-configured nudge parameter (in minutes) representing the duration of app blocking once the consecutive usage threshold is reached.
- Daily goal selection: An optional, self-selected daily intention chosen during the check-in (Chill, Work, Study, or Skipped). Purely indicative and does not alter nudge parameters. Treated as an in-app behavioral indicator of goal selection patterns, distinct from traditional self-report, because it is a repeated contextual choice reflecting immediate priorities rather than retrospective attitudes.

Usage-triggered nudge logs captured “nudge reached” events—the date and time at which users’ consecutive usage exceeded their configured threshold, triggering app blocking. DSCT configuration logs recorded updates to nudge parameters (consecutive usage threshold and cooldown length), monitored app selections, and monitored interactions with manual app blocking features. Daily check-in completions, including the user’s daily goal selection, were recorded with associated timestamps. Together, these data sources provided the behavioral indicators used for RQ1 (user-nudge interaction ratio over time), RQ2 (configuration divergence and spillover effects), and RQ3 (preintervention behavioral differences between subgroups).

Self-reported data were gathered through multiple modalities:

1. Onboarding surveys: Participants completed assessments of their device usage patterns, including daily screen time, intended screen time goals, frequency of regretted scrolling sessions, and contexts in which such regret occurred. These measures, collected at app installation, serve as traditional self-reported baseline indicators (used for RQ3).
2. Postintervention survey: A single-choice questionnaire capturing participants’ rationale for accepting or rejecting the reconfiguration prompt (used for RQ3).

Demographic data were collected through multiple methodological approaches: age and gender data were obtained via DSCT onboarding surveys, and geographical location was determined through Mixpanel analytics tools.

Related to the nudge reconfiguration prompt, experimental response data documented the temporal sequence of intervention-related events with precise timestamps. The platform recorded intervention acceptance or rejection, subsequent nudge settings adjustments, and postintervention interaction patterns.

The daily engagement framework, facilitated by the mandatory daily check-in feature, ensured consistent participation and enabled measurement of daily user interaction and attrition rates. Together, these mechanisms established a robust environment for examining variations in user interaction and self-regulation outcomes.

### Data Analysis

We structured our analyses around the 3 research questions.

For RQ1, we computed the daily user-nudge interaction ratio for each group and compared 7-day preintervention (November 16‐22, 2024) and postintervention (November 24‐30, 2024) averages across the acceptance subgroup, rejection subgroup, and control group. The preintervention and postintervention windows were selected to maximize temporal separation from the rolling enrollment period (November 22‐24, 2024) while maintaining 7-day averaging; minor overlap on boundary days was mitigated by aggregation. To assess whether the acceptance subgroup change was attributable to the intervention rather than temporal trends, we applied a difference-in-differences (DiD) analysis at the daily aggregate level, using permutation tests (10,000 iterations) to derive *P* values. This approach compares each subgroup’s pre-to-post change against the control group’s change.

For RQ2, we analyzed the time-series of the user-nudge interaction ratio across a 5-week window (1 week preintervention and 4 weeks postintervention) to assess the sustainability of re-engagement. We compared DSCT configuration parameters (consecutive usage threshold and cooldown length distributions) and manual app blocking interactions between the acceptance and rejection subgroups at 2 time points (preintervention: November 20, 2024; postintervention: November 29, 2024) using independent-samples *t* tests with Cohen *d* as a measure of effect size.

For RQ3, we compared 2 categories of indicators between the acceptance and rejection subgroups: (1) observable behavioral indicators—consecutive usage threshold and cooldown length (independent-samples *t* tests) and daily goal selection coded as binary leisure versus nonleisure (chi-square test with Cramer *V*)—and (2) traditional self-reported measures—onboarding survey responses on screen time goals, scrolling regret frequency, and emotional responses (independent-samples *t* tests) and postintervention survey responses. By comparing the statistical significance of behavioral indicators against self-reported measures, we assessed which category better differentiates users who accept from those who reject the reconfiguration prompt.

All statistical analyses were conducted by exporting Mixpanel data into a Jupyter Notebook environment. The study applied a statistical significance threshold of *P*<.05. All *t* tests were 2-tailed. Effect sizes were calculated using Cohen *d* for continuous variables and Cramer *V* for categorical comparisons.

### Ethical Considerations

This study was considered a service evaluation of a commercially available app, analyzing anonymized and nonidentifiable in-app usage data. As such, it was not subject to the requirements for formal ethics committee approval, consistent with institutional guidelines for nonclinical research involving deidentified data. No personally identifiable information was collected, and participants could not be reidentified by researchers due to the app’s tokenization system, which is compliant with Apple’s privacy guidelines for app monitoring. Participants, or their legal representatives in the case of minors, consented to the use of their anonymized data for research purposes through the app’s terms of service and onboarding process. All data were stored and processed in compliance with applicable privacy regulations, and no compensation was provided to participants.

## Results

### Participant Characteristics

Analysis of participant demographics (Table 1) confirmed alignment with the broader DSCT user base. The sample was predominantly young, with 51.4% (71/138) of experimental participants and 57% (65/114) of control participants aged 18 to 24 years. Among the 137 (54.4%) participants who reported gender, 86 (62.8%) identified as male. Participants were geographically distributed across 55 countries, with the largest concentrations in North America and Europe. No significant demographic differences were found between the experimental and control groups (all *P*>.05; Table 1), validating the initial randomization. Demographic distributions were also consistent between the acceptance and rejection subgroups within the experimental condition (age: *P*=.68; gender: *P*=.33; geography: *P*=.19), demonstrating that demographic characteristics did not influence participants’ decisions to accept or reject the prompt.

**Table 1.** Demographic characteristics of study participants by group assignment.^a^

Characteristic	Experimental (n=138), n (%)	Control (n=114), n (%)	*P* value^b^
Age (y)			.24
<18	38 (27.5)	25 (21.9)	
18‐24	71 (51.4)	65 (57)	
25‐34	16 (11.6)	13 (11.4)	
≥35	13 (9.4)	4 (3.5)	
Gender^c^			.16
Male	42 (30.4)	44 (38.6)	
Female	21 (15.2)	26 (22.8)	
Other or prefer not to say	0 (0)	4 (3.5)	
Not reported	75 (54.3)	40 (35.1)	
Geographic region^d^			.30
North America	38 (27.5)	37 (32.5)	
Europe	48 (34.8)	42 (36.8)	
Asia-Pacific	25 (18.1)	22 (19.3)	
Middle East and Africa	22 (15.9)	8 (7)	
Latin America	5 (3.6)	5 (4.4)	

a The experimental (n=138) and control (n=114) groups showed no significant differences in age, gender, or geographic distribution (all *P*>.05), confirming successful randomization.

b *P* values from chi-square tests comparing the experimental and control groups. The gender test excludes participants who did not report gender. Age data were unavailable for 7 control participants due to nonnumeric entries in the analytics platform.

c Gender data were collected via an optional onboarding survey item. Among the 137 out of 252 (54.4%) participants who reported gender, 86 (62.8%) identified as male, 47 (34.3%) as female, and 4 (2.9%) as other or preferred not to say.

d Geographic region determined via app analytics geolocation.

### RQ1: Intervention Effect on User-Nudge Interaction

#### Overview

**Figure 3.**
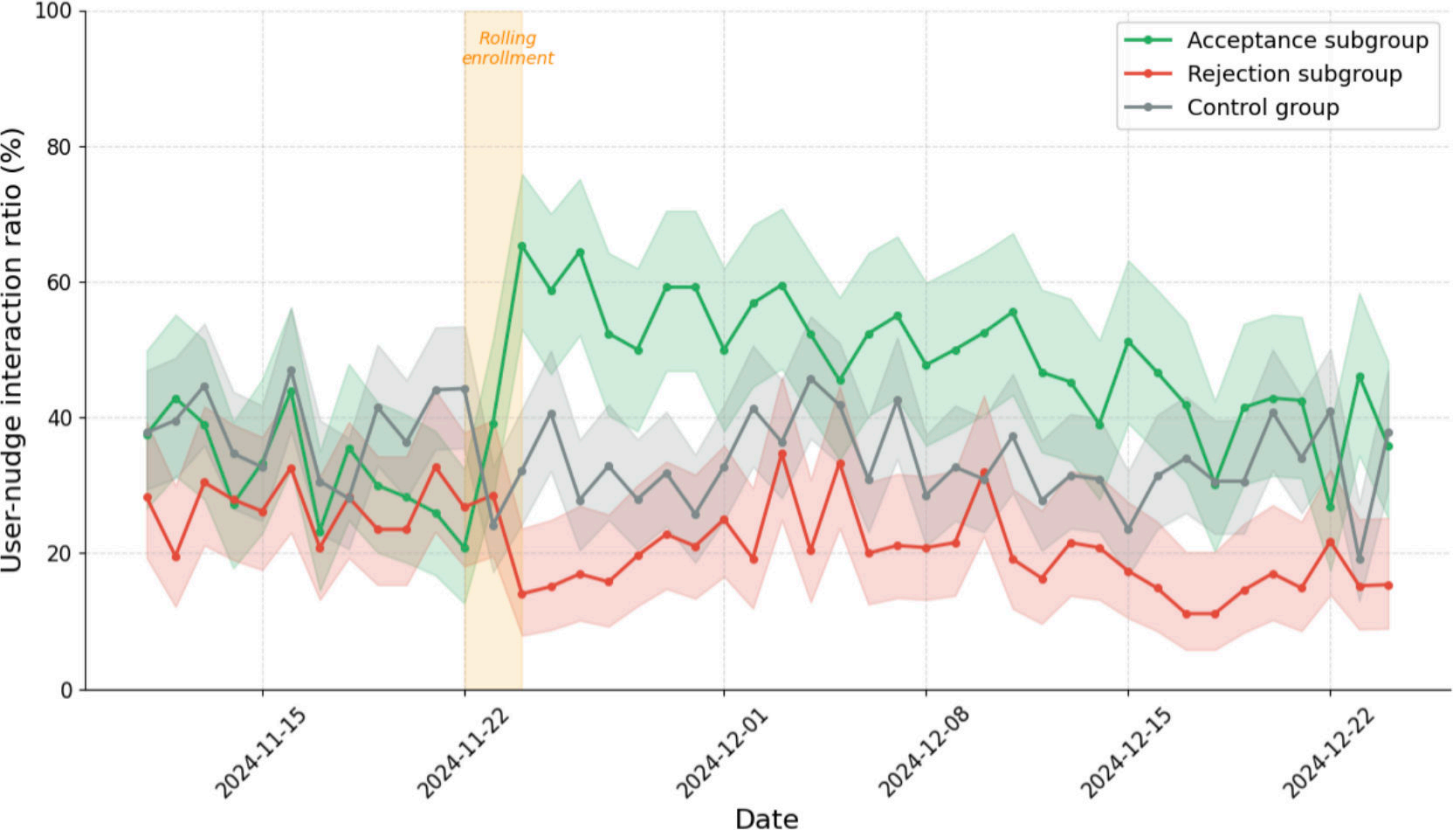
Temporal evolution of the daily user-nudge interaction ratio (%) across the acceptance subgroup (n=63), rejection subgroup (n=75), and control group (n=114). Shaded bands represent 95% Wilson score CIs. The orange band marks the rolling enrollment window (November 22‐24, 2024). Postintervention, the acceptance subgroup’s 7-day average rose from 29.7% to 58.5%, whereas both the rejection subgroup and control group showed declines.

To assess the intervention’s effect on user-nudge interaction, we compared 7-day preintervention and postintervention averages and applied a DiD analysis to control for temporal trends (see the Methods section). Of the 138 experimental participants, 46% (n=63) accepted the nudge reconfiguration prompt, yielding 3 cohorts: the acceptance subgroup (n=63), rejection subgroup (n=75), and control group (n=114). Figure 3 illustrates the temporal evolution of the daily user-nudge interaction ratio, and Table 2 summarizes the DiD results.

**Table 2.** Difference-in-differences analysis of user-nudge interaction ratio (7-day preintervention and postintervention averages).^a^

Comparison	Subgroup change (pp^b^)	Control change (pp)	Difference-in-differences (pp)	*P* value
Acceptance vs control	+28.8	−7.5	+36.3	<.001
Rejection vs control	−9	−7.5	−1.4	.82

a The acceptance subgroup’s increase was significant after controlling for the temporal decline observed in the control group (difference-in-differences=+36.3 pp, *P*<.001), whereas the rejection subgroup’s decline was not significantly different from that of the control group (difference-in-differences=−1.4 pp, *P*=.82), confirming the intervention was not counterproductive.

b pp: percentage points.

#### Acceptance Subgroup Outcomes

The acceptance subgroup’s 7-day average user-nudge interaction ratio increased from 29.7% preintervention to 58.5% postintervention (peak of 65% on day 1). This increase remained significant after controlling for the general temporal decline observed in the control group (Table 2).

#### Rejection Subgroup Outcomes

The rejection subgroup’s 7-day average declined from 26.9% to 17.9%. This decline was not significantly different from that of the control group’s decline (Table 2), confirming that the intervention was not counterproductive for users who declined the prompt. Throughout the observation period, this subgroup consistently demonstrated the lowest engagement rates across all cohorts.

#### Control Group Performance

The control group’s 7-day average declined from 38.8% to 31.3%, consistent with a general temporal trend unrelated to the intervention. The acceptance subgroup’s interaction ratio converged with the control group’s, approximately 1 month post intervention.

### RQ2: Temporal Evolution and Behavioral Divergence

#### Overview

Having established the significance of the intervention effect (RQ1), we examined the sustainability of re-engagement over time. Figure 3 illustrates the temporal evolution of the user-nudge interaction ratio across the 5-week observation window. The acceptance subgroup elevated engagement persisted above baseline for approximately 2 weeks before gradually declining, converging with control group levels by the 1-month mark. The rejection subgroup sustained the lowest engagement across all cohorts throughout the observation period.

#### Nudge Settings Configuration

**Figure 4.**
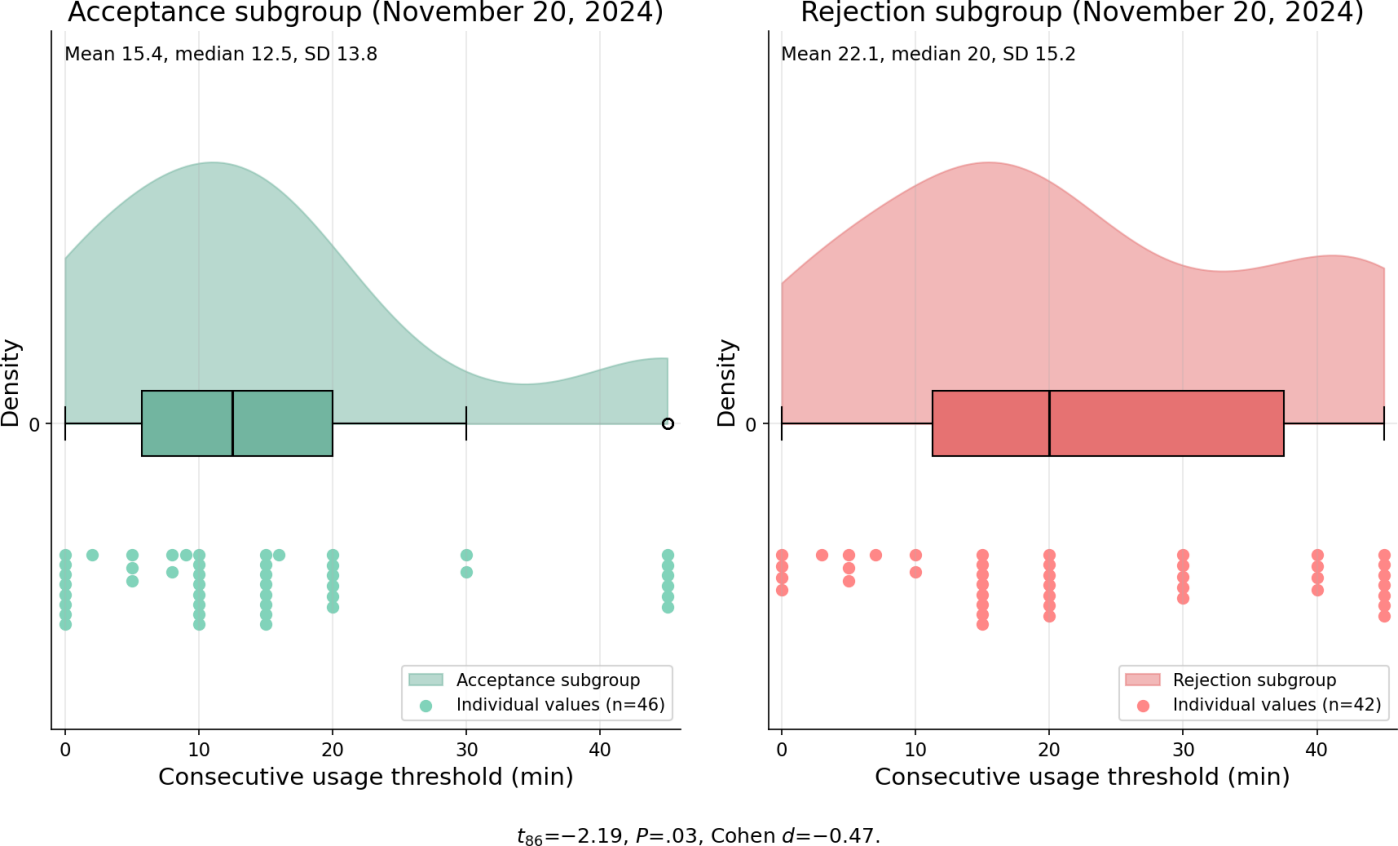
Distribution of consecutive usage threshold (min) between the acceptance subgroup (n=63) and rejection subgroup (n=75) on November 20, 2024 (preintervention). Acceptance subgroup participants configured 21.53% shorter consecutive usage thresholds (*t*_86_=−2.19, *P*=.03, Cohen *d*=−0.47). Individual values reflect participants with active nudge configurations on the indicated date.

Three days prior to the reconfiguration prompt, the acceptance subgroup configured shorter consecutive usage thresholds (−21.53%) and longer cooldown periods (+20.56%) compared with the rejection subgroup. The consecutive usage threshold disparity widened significantly 1 week post prompt, from *t*_86_=−2.19, *P*=.03, Cohen *d*=−0.47 to *t*_98_=−3.28, *P*=.002, Cohen *d*=−0.67, as depicted in Figures4  5 (data from November 20 and 29, 2024). The cooldown length difference was not statistically significant at either time point (pre-intervention: t₈₆=0.62, P=.54, Cohen d=0.13; post-intervention: t₉₈=−0.08, P=.93, Cohen d=−0.02). Acceptance subgroup participants largely maintained their reconfigured nudge settings (median=10 min) post intervention.

**Figure 5.**
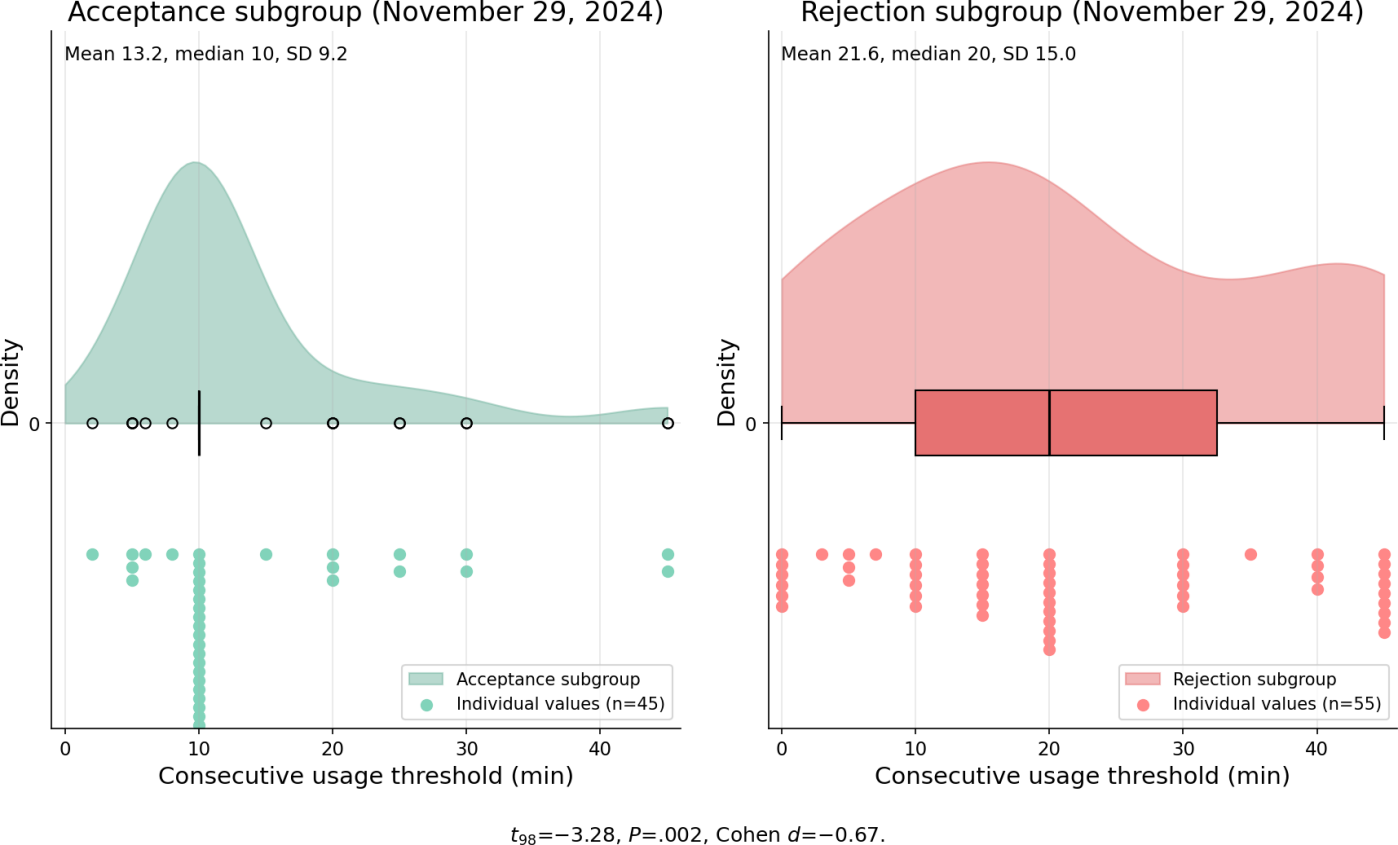
Distribution of consecutive usage threshold (min) between the acceptance subgroup (n=63) and rejection subgroup (n=75) on November 29, 2024 (postintervention). The effect size widened from Cohen *d*=−0.47 to Cohen *d*=−0.67 (*t*_98_=−3.28, *P*=.002). Individual values reflect participants with active nudge configurations on the indicated date.

#### Manual App Blocking Interaction

Although the reconfiguration prompt targeted nudge settings rather than manual app blocking, the distribution of manual app blocking interactions between the acceptance and rejection subgroups diverged during the postintervention observation period. This divergence increased from November 20 (*t*_86_=1.82, *P*=.07, Cohen *d*=0.39) to November 29 (*t*_98_=2.58, *P*=.01, Cohen *d*=0.49), as shown in Figure S2 in Multimedia Appendix 1.

### RQ3: Behavioral Indicators Versus Self-Reported Measures

#### Overview

The behavioral divergence reported under RQ2, particularly in consecutive usage threshold (*P*=.03) and, to a lesser extent, cooldown length (*P*>.05), suggested that observable DSCT configuration differences predate the intervention. To determine whether such in-app behavioral indicators are more strongly associated with intervention acceptance than traditional self-reported measures, we compared both categories of indicators between the acceptance and rejection subgroups, as described in the Data Analysis section.

#### Daily Check-in Goals

Compared with the rejection subgroup, acceptance subgroup participants demonstrated a significantly lower tendency to select “Chill” (leisure) as a daily goal (15.6% vs 26.2%; chi-square *P*=.001, Cramer *V*=0.13), as illustrated in Figure S3 in Multimedia Appendix 1. This pattern was stable across the observation period, showing no preintervention or postintervention change.

#### Onboarding Survey Analysis

Analysis of onboarding survey responses regarding current screen time, and screen time reduction goals revealed no significant differences (*P*>.1) between acceptance and rejection subgroups, as depicted in Figures S4 and S5 in Multimedia Appendix 1.

Further analysis of responses regarding scrolling regret frequency and associated emotional responses also yielded no significant differences (*P*>.1) between groups, as shown in Figures S6 and S7 in Multimedia Appendix 1. While response patterns were similar, both distributions exhibited comparable trends, evidenced by the similar distribution of the “Disappointed” response for scrolling-associated emotions.

#### Postintervention Survey

Postintervention survey data showed no statistically significant differences between the acceptance and rejection subgroups (*P*>.05), consistent with the pattern observed for other self-reported measures in this study.

## Discussion

### RQ1: Effectiveness of the Reconfiguration Prompt

The 46% (63/138) acceptance rate for nudge reconfiguration among passive users indicates that nearly half possess latent readiness to re-engage with DSCTs when prompted. The DiD analysis confirms that the acceptance subgroup’s increase in user-nudge interaction (DiD=+36.3 percentage points, *P*<.001) is attributable to the intervention rather than temporal trends, as the rejection subgroup’s decline was not significantly different from the control group’s decline (DiD=−1.4 percentage points, *P*=.82). The comparable preintervention baselines between subgroups further support the conclusion that the observed change is driven by the intervention itself.

### RQ2: Temporal Dynamics and Behavioral Divergence

The preintervention behavioral differences between subgroups are particularly noteworthy. Acceptance subgroup participants configured 21.53% shorter consecutive usage thresholds and descriptively longer cooldown periods (+20.56%, though not statistically significant) compared with the rejection subgroup, even before receiving the intervention. The widening of the consecutive usage threshold gap postintervention (Cohen *d* increasing from −0.47 to −0.67) indicates that the prompt amplified existing behavioral differences rather than creating new ones. The spillover effect observed in manual app blocking behaviors (Cohen *d* increasing from 0.39 to 0.49) further suggests that successful re-engagement with one DSCT feature may catalyze broader self-regulatory behaviors. However, the gradual decline in user-nudge interaction after approximately 2 weeks, converging with control group levels by 1 month, demonstrates the limitations of single-prompt interventions and aligns with SDT: extrinsic prompts alone cannot sustain behavioral change without corresponding intrinsic motivation [24].

### RQ3: The Intention-Behavior Gap

The most striking finding is the disconnect between self-reported measures and behavioral indicators. Despite no significant differences in stated screen time goals, scrolling regret frequency, or emotional responses between the acceptance and rejection subgroups (all *P*>.1), observable behavioral indicators—shorter consecutive usage thresholds (*P*=.03) and lower selection of leisure-oriented daily goals (*P*=.001)—clearly differentiated those who would accept the reconfiguration prompt. Even the postintervention survey, which directly captured participants’ rationales, showed no significant differences (*P*>.05). This intention-behavior gap [35] underscores the limitations of relying on self-reported data to characterize DSCT engagement and highlights the value of DSCT configuration logs and in-app behavioral choices as proxies for readiness to change.

### Implications for DSCT Design and Implementation

Our findings challenge conventional approaches to DSCT user engagement. The 46% (63/138) acceptance rate among passive users and subsequent boost in user-nudge interaction challenge the binary classification of users as either engaged or disengaged, suggesting instead that engagement exists on a continuum with opportunities for reactivation even among seemingly passive users. This perspective encourages DSCT developers to implement proactive re-engagement strategies rather than simply accepting attrition as inevitable.

These proactive re-engagement strategies have proven more effective for users with higher readiness to change. Our preintervention data revealed that acceptance subgroup participants configured significantly shorter consecutive usage thresholds (−21.53%, *P*=.03), with a directionally consistent but nonsignificant difference in cooldown length (+20.56%), indicating that observable DSCT configuration behaviors are significantly associated with intervention receptiveness. DSCTs aiming to offer personalized experiences should leverage such behavioral markers to identify users most likely to benefit from re-engagement prompts, thereby optimizing intervention effectiveness.

However, the temporary nature of increased engagement reveals a critical limitation. While our intervention successfully boosted the acceptance subgroup’s 7-day average user-nudge interaction ratio from 29.7% to 58.5% (peak of 65% on day 1), convergence with the control group after approximately 1 month demonstrates that single-prompt interventions alone cannot sustain behavioral change. Sustainable engagement requires progressive feature designs that transition users from external prompts to self-directed regulation, perhaps through adaptive goal setting, competence-building feedback, and autonomy-supportive interfaces.

Throughout these design considerations, the preservation of user autonomy emerges as a fundamental principle. DSCTs must navigate the inherent tension between providing behavioral support and avoiding paternalistic control. This delicate balance requires thoughtful intervention framing, opt-in mechanisms, customizable parameters, and transparent communication about the rationale behind nudges and restrictions. Only by respecting user agency can DSCTs foster the intrinsic motivation necessary for lasting behavior change, as our divergent group outcomes clearly demonstrate.

### Limitations

This study has several limitations that inform the interpretation of our findings. Although the DiD analysis strengthens causal inference by controlling for temporal trends shared across groups, our quasi-experimental design cannot fully rule out self-selection effects, as preexisting behavioral differences between acceptance and rejection subgroups—particularly in nudge configurations—suggest self-selection dynamics [36] that complicate attribution of outcomes solely to the intervention. While these baseline differences provide valuable insights about readiness indicators, they prevent definitive conclusions about intervention causality.

Beyond design constraints, our reliance on behavioral proxies for psychological constructs introduces measurement limitations. Although usage patterns and configuration choices offer objective indicators, they cannot fully capture the complexity of readiness to change. Moreover, onboarding survey data used to assess baseline attitudes may not reflect participants’ psychological states at the time of the experiment, as these surveys were collected at app installation, which for some participants occurred months before study enrollment.

Additionally, generalizability constraints arise from our sample characteristics. Participants were drawn exclusively from a single DSCT (Detox) with a specific design philosophy, predominantly young users (with over half aged 18‐24 y and approximately one-quarter aged <18 y), and geographic concentration in North America and Europe. These factors may limit the applicability of our findings to other DSCTs, age groups, or cultural contexts in which digital self-regulation practices differ. An important point to note is that our sample consisted exclusively of users who voluntarily installed a DSCT to reduce screen time, suggesting higher baseline motivation than the general population. This limits generalizability to individuals who have not actively sought digital self-regulation tools.

Furthermore, we cannot determine whether the decline in user-nudge interaction observed in Figure 3 represents a successful change in behavior that reduces reliance on the tool or disengagement from self-regulation efforts, as we lack direct measures of participants’ real-world digital well-being behaviors outside the DSCT environment.

### Future Research Directions

Our findings illuminate critical avenues for advancing DSCT effectiveness research. The convergence of acceptance and control groups after 1 month raises fundamental questions about intervention durability. This pattern suggests that single-prompt interventions have limited sustainability, pointing toward the need for longitudinal studies investigating optimal intervention cadences. Research should test whether periodic re-engagement at empirically determined intervals can maintain elevated user engagement without inducing prompt fatigue.

Building on our identification of behavioral readiness indicators, predictive modeling represents a promising research direction. While our study identified several behavioral indicators associated with acceptance, comprehensive models incorporating temporal patterns, contextual factors, and usage trajectories could enable dynamic, personalized intervention timing. Machine learning approaches could identify complex behavioral signatures that signal optimal intervention moments, moving beyond static threshold rules to recognize when users are most receptive to change. Future research may integrate comprehensive psychological assessments, ecological momentary assessments of human-device interaction, objective and longitudinal measures of smartphone and app use (eg, usage logs), as well as contextual data on physical and social environments to better understand how these factors jointly influence digital well-being.

Furthermore, comparative effectiveness research across theoretical frameworks is essential for establishing evidence-based DSCT design principles. Our study examined 1 SDT-based approach, but the field would benefit from systematic comparisons of different intervention philosophies. Understanding which approaches work best for different user segments, cultural contexts, and problematic usage patterns could guide the development of more personalized and effective interventions.

These research directions collectively aim to transform DSCTs from static tools to adaptive systems that recognize and respond to users’ evolving motivational states while preserving the autonomy essential for lasting behavioral change.

### Conclusion

This study demonstrates that prompting passive DSCT users to reconfigure their nudge settings can effectively restore engagement (RQ1), with effects persisting for approximately 1 month before converging with baseline levels (RQ2). Observable behavioral indicators—including shorter consecutive usage thresholds (*P*=.03) and lower selection of leisure-oriented daily goals (*P*=.001)—differed significantly between participants who accepted versus rejected the intervention, whereas self-reported measures showed no such differences (RQ3). This intention-behavior gap underscores the value of in-app behavioral data over self-report measures for understanding user engagement with digital well-being interventions.

Our findings suggest that the future of effective DSCT design lies not in creating more restrictive or feature-rich tools but in developing adaptive systems tailored to the needs of specific population clusters and based on SDT principles that recognize and respond to users’ motivational states while preserving their autonomy, increasing their competence and reinforcing relatedness. As digital well-being becomes an increasingly critical public health concern, understanding the psychological mechanisms underlying successful self-regulation in digital contexts becomes essential for developing interventions that create lasting positive change.

## Supplementary material

10.2196/85349 Multimedia Appendix 1 Detailed nudge reconfiguration settings, participant inclusion criteria, and supplementary statistical analyses.

## Abbreviations

DiD: difference-in-differences
DSCT: digital self-control tool
RQ: research question
SDT: self-determination theory
