# Supplementary material for: Restoring Engagement in Digital Self-Control Tools Using Nudge Reconfiguration Prompts: Quasi-Experimental Study
Source: JMIR Form Res. 2026 Apr 28;10:e85349. doi: 10.2196/85349 (PMC13123756; doi:10.2196/85349)
Supplement: Multimedia Appendix 1 [file formative-v10-e85349-s001.pdf]

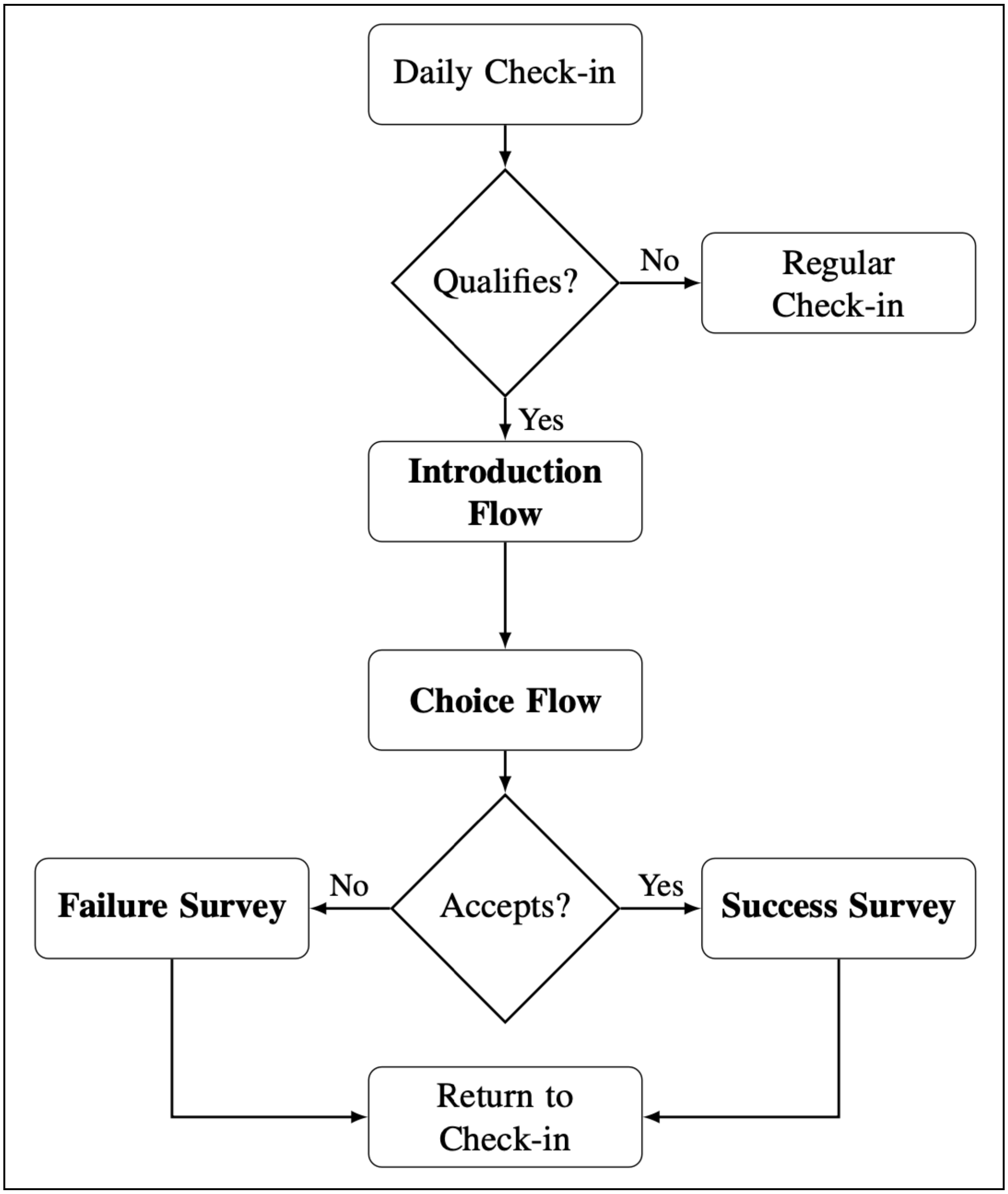

Figure S1. Study design flowchart. Participants (n=252) were randomly assigned to the experimental group (n=138) or the control group (n=114). Experimental group participants received a reconfiguration prompt during their daily check-in. Based on their response, they were classified into the acceptance subgroup (n=63) or the rejection subgroup (n=75).

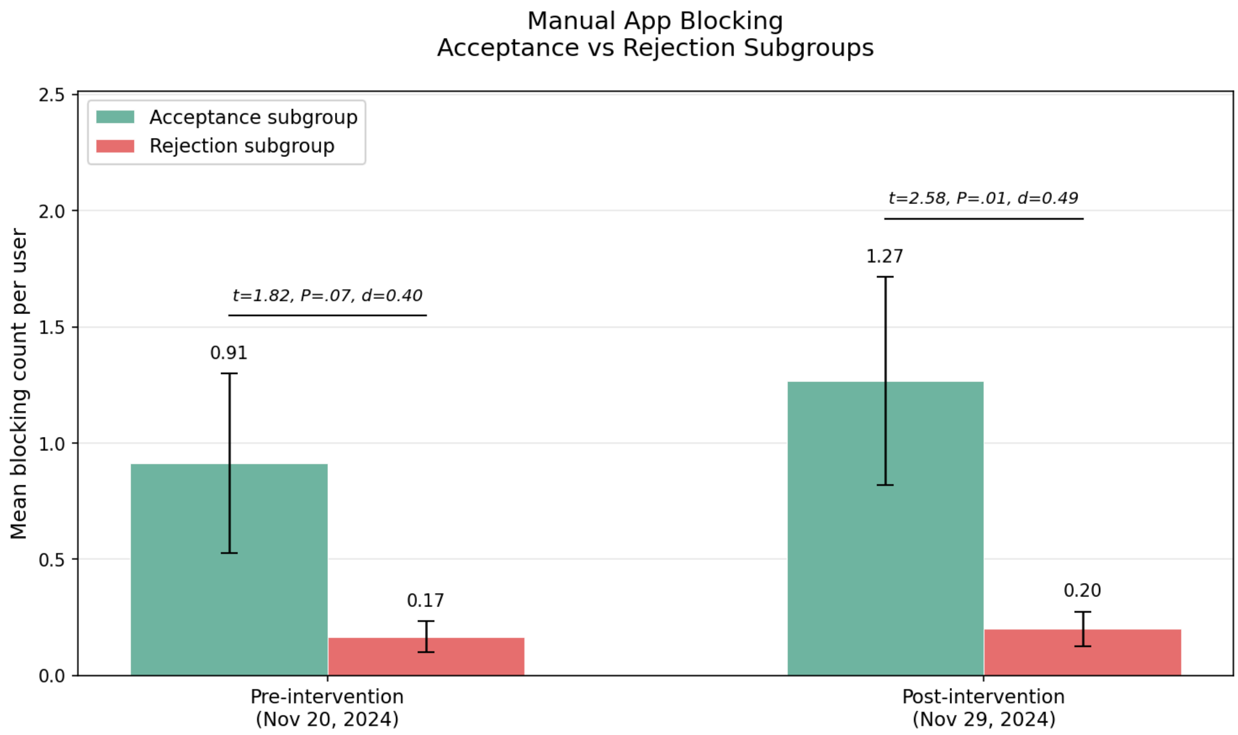

Figure S2. Distribution of manual app blocking counts for the acceptance subgroup and rejection subgroup at two time points: pre-intervention (November 20, 2024; left panel) and post-intervention (November 29, 2024; right panel). Box plots show medians and interquartile ranges; individual data points are overlaid with jitter. The divergence widened from t=1.82, P=.07, d=.40 (pre) to t=2.58, P=.01, d=.49 (post).

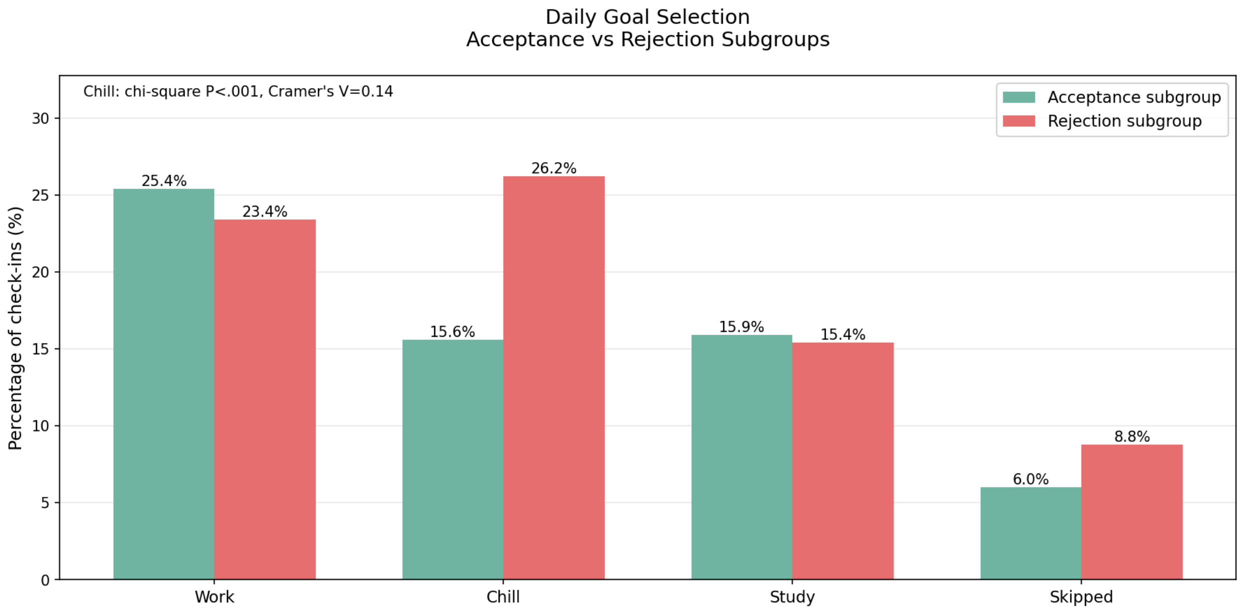

Figure S3. Distribution of daily goal selection (Work, Chill, Study, or Skipped) for the acceptance subgroup and rejection subgroup during the pre-intervention period (November 15-22, 2024). The acceptance subgroup selected "Chill" (leisure) significantly less frequently than the rejection subgroup (15.6% vs 26.2%; chi-square P<.001, Cramer's V=.14).

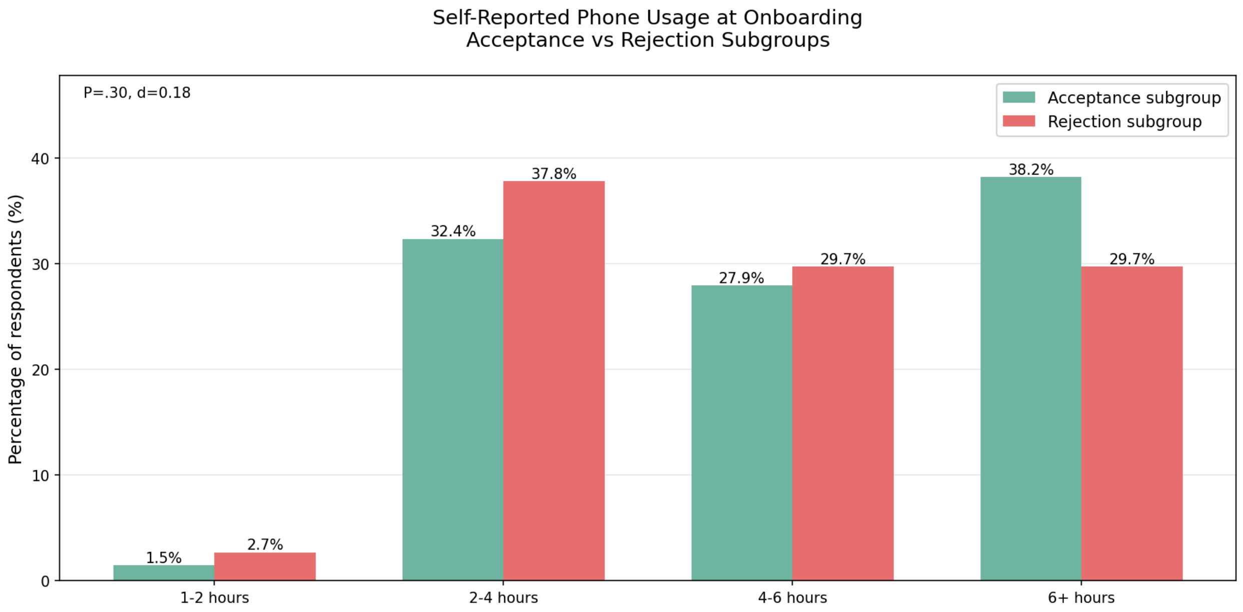

Figure S4. Self-reported phone usage at onboarding for the acceptance subgroup and rejection subgroup. No significant difference was observed (P=.30).

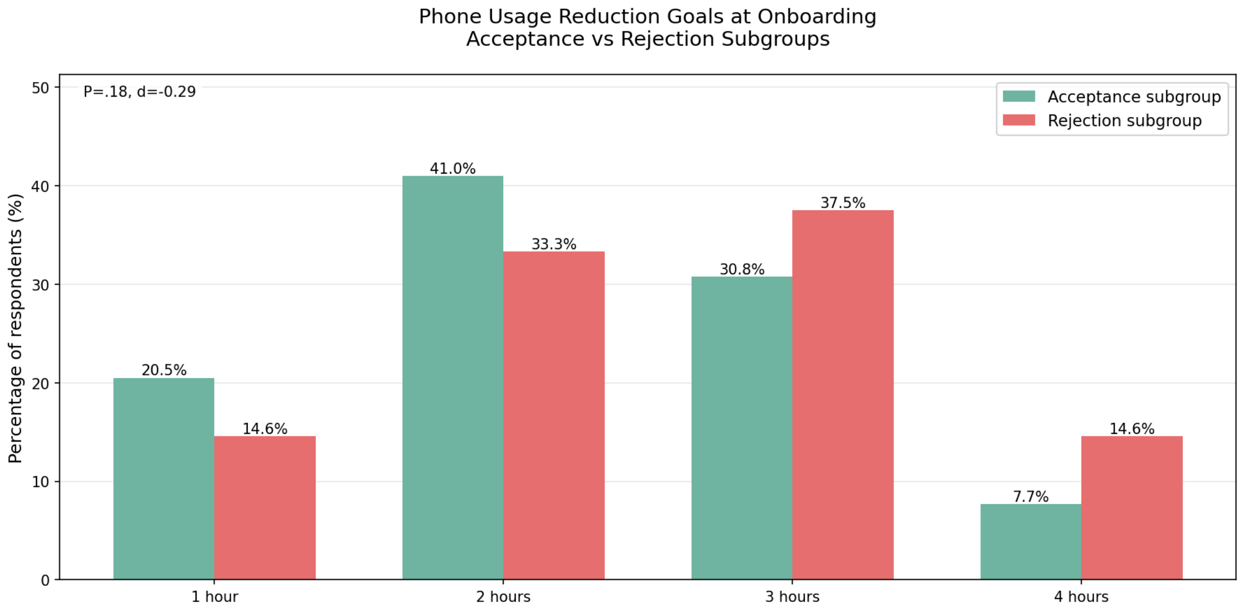

Figure S5. Phone usage reduction goals at onboarding for the acceptance subgroup and rejection subgroup. No significant difference was observed (P=.18).

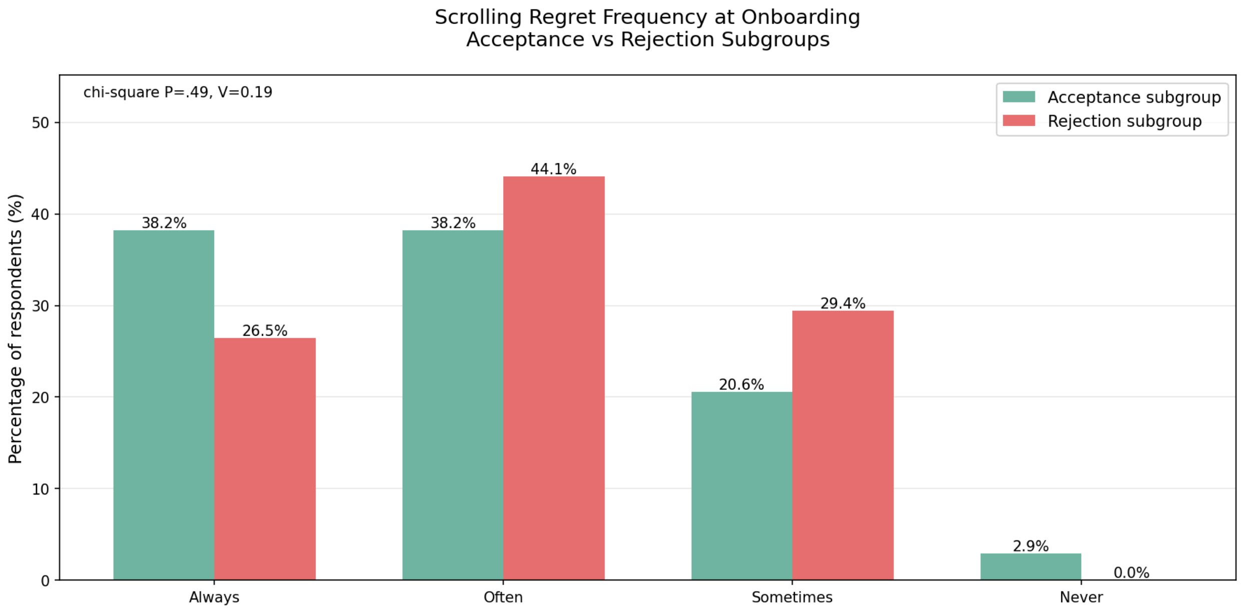

Figure S6. Scrolling regret frequency at onboarding for the acceptance subgroup and rejection subgroup. No significant difference was observed (chi-square P=.49).

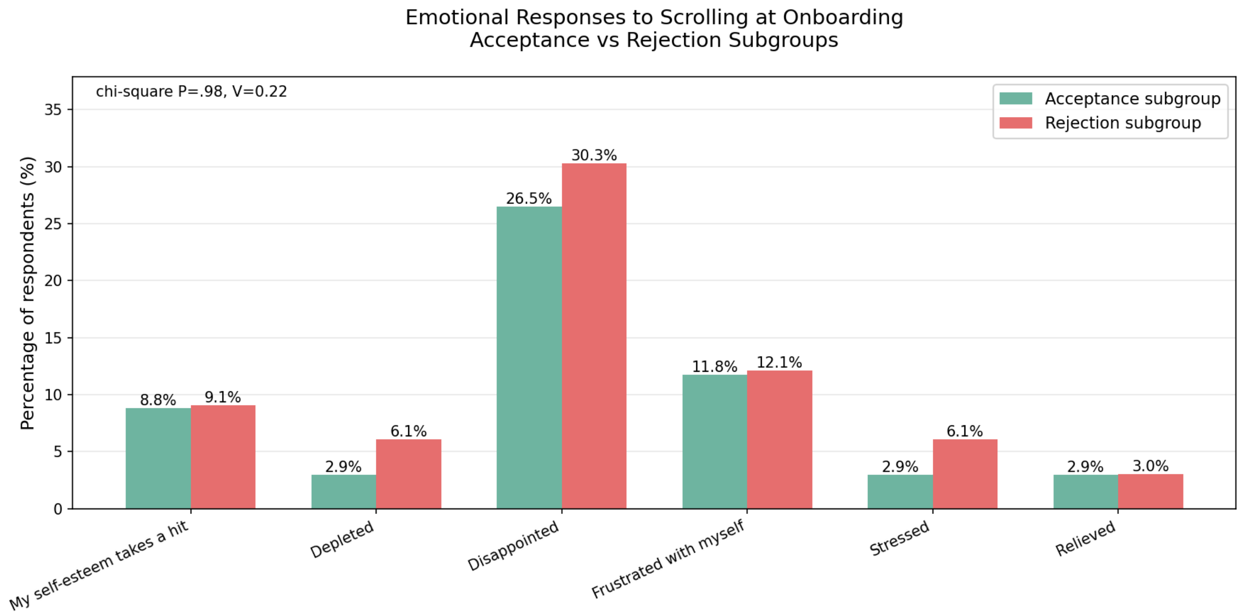

Figure S7. Emotional responses to scrolling at onboarding for the acceptance subgroup and rejection subgroup. No significant difference was observed (chi-square P=.98).
